# Association of Emotion, Sleep Quality With Hypertension and Complications in the Elderly Population

**DOI:** 10.1002/brb3.70676

**Published:** 2025-07-07

**Authors:** Jin Wang, Yunwei Zhang, Yiman Feng, Ya Yang, Hansheng Ding

**Affiliations:** ^1^ Shanghai Health Development Research Center (Shanghai Medical Information Center) Shanghai P.R. China; ^2^ Dahua Hospital Shanghai China

**Keywords:** complications, elderly population, emotional state, hypertension, sleep quality

## Abstract

**Introduction:**

Hypertension is a highly prevalent chronic disease in the elderly population, often accompanied by serious complications, such as heart disease, stroke, and kidney failure. The present study aimed to explore the association of emotional state and sleep quality with hypertension and its complications in the elderly population.

**Methods:**

This cross‐sectional study was conducted in Shanghai, China, and included a total of 168,860 elderly participants, of whom 97,565 had hypertension and 65,454 had complications. Data on demographic characteristics, emotional status, sleep quality, and disease conditions were collected using the Unified Needs Assessment Form for Elderly Care Questionnaire. Multivariate logistic regression was performed to analyze the data, with a particular focus on the presence of complications.

**Results:**

The results indicated that demographic variables such as age, sex, and lifestyle factors (including diet and body mass index) were significantly associated with hypertension and its complications. Hypertensive patients, particularly those with complications, exhibited significantly higher levels of emotional instability (such as irritability and fatigue) and poor sleep quality compared to non‐hypertensive individuals. Multivariate logistic regression analysis demonstrated that emotional fluctuations and poor sleep quality were positively related to hypertension and complications.

**Conclusions:**

The results suggested that emotional health and sleep quality are important factors associated with hypertension and complications in the elderly. These findings underscore the need for comprehensive management strategies that address both psychological and sleep‐related factors to improve hypertension control and prevent associated complications.

## Graphical abstract

The present study suggested that emotional health and sleep quality were are important factors associated with hypertension and complications in the elderly. These findings underscored the need for comprehensive management strategies that address both psychological and sleep‐related factors, aiming to improve hypertension control and prevent associated complications

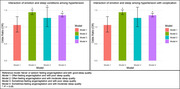

## 1 Introduction

Hypertension is one of the most common chronic diseases threatening older adults and is a significant contributor to the global health burden (Yang et al. 2025). The prevalence of hypertension among adults aged 60 and older is about 54.6% in China and 63.1% in the United States (Liu et al. 2018; National Center for Health Statistics 2017). If left uncontrolled, hypertension can lead to serious complications, including heart disease, stroke, and kidney disease (Oliveros et al. 2018; Zhou et al. 2021). The association of pulse pressure with all‐cause and cause‐specific mortality has been shown to be significant, with a notable proportion of deaths attributed to elevated systolic blood pressure occurring in adults under the age of 70 (Liu et al. 2021; World Health Organization 2023). Besides these mortality outcomes, the development and progression of hypertension are shaped by a variety of risk factors throughout the lifespan. According to the Guidelines for Prevention and Treatment of Hypertension in China (2024) (Chinese Hypertension League 2024), these include age, sex, educational attainment, high dietary sodium intake, and physical inactivity (Hu et al. 2017; Maksimova and Maksimov 2019; Ma et al. 2021; Cao et al. 2019).

Recent research increasingly shows that hypertension in older adults is closely associated with emotional and sleep‐related disturbances. For example, hypertension is associated with a higher likelihood of developing depressive symptoms, with an odds ratio (OR) of 1.28 (Cai et al. 2022; Qiu et al. 2024). Other psychological conditions, including chronic psychological stress, post‐traumatic stress disorder (PTSD) (hazard ratios, HRs: 1.46–3.28), and anxiety (HR: 1.26), are also positively associated with elevated blood pressure (Cohen et al. 2015). Similarly, sleep‐related issues such as obstructive sleep apnea (OSA) and a high oxygen desaturation index (ODI) have been shown to significantly increase the risk of hypertension (OR: 1.94) (Han et al. 2020). Furthermore, inadequate sleep duration (OR: 1.20) and unhealthy sleep patterns (OR: 1.90) have also been identified as significant risk factors for hypertension development (Li and Shang 2021; Haghayegh et al. 2023).

While the associations between hypertension and psychosocial or sleep‐related factors have been explored in various studies, limited attention has been paid specifically to elderly populations, particularly within the Chinese context. Elderly individuals in China often experience unique stressors, such as intergenerational caregiving burdens, limited access to sleep disorder screening, and a high prevalence of untreated emotional disorders. These factors may collectively contribute to a heightened risk of hypertension and related complications (Wu et al. 2024; Zhao and Zheng 2024; Huang 2024). Although some literature suggests that lifestyle modifications, such as improving sleep quality, may help prevent complications like heart failure (Di Palo and Barone 2020), large‐scale quantitative evidence targeting older adults in China remains insufficient (Lu et al. 2015; Jiang et al. 2023). This highlights a critical research gap regarding how emotional and sleep‐related factors interact with hypertension complications in aging Chinese populations.

## 2 Methods

### 2.1 Study Design

This study was based on a cross‐sectional survey, involving elderly individuals aged 60 and above who voluntarily applied for and received the Elderly Care Unified Needs Assessment for Long‐Term Care Insurance in Shanghai, China, between January and May 2023. The research protocol, bearing the reference number 2024002, obtained official approval from the Ethics Committee of the Shanghai Health Development Research Center. Before the assessment began, all applicants signed an informed consent form. For elderly individuals who were too frail or unable to sign, a legal guardian was permitted to sign on their behalf.

### 2.2 Inclusion Criteria

**FIGURE 1:**
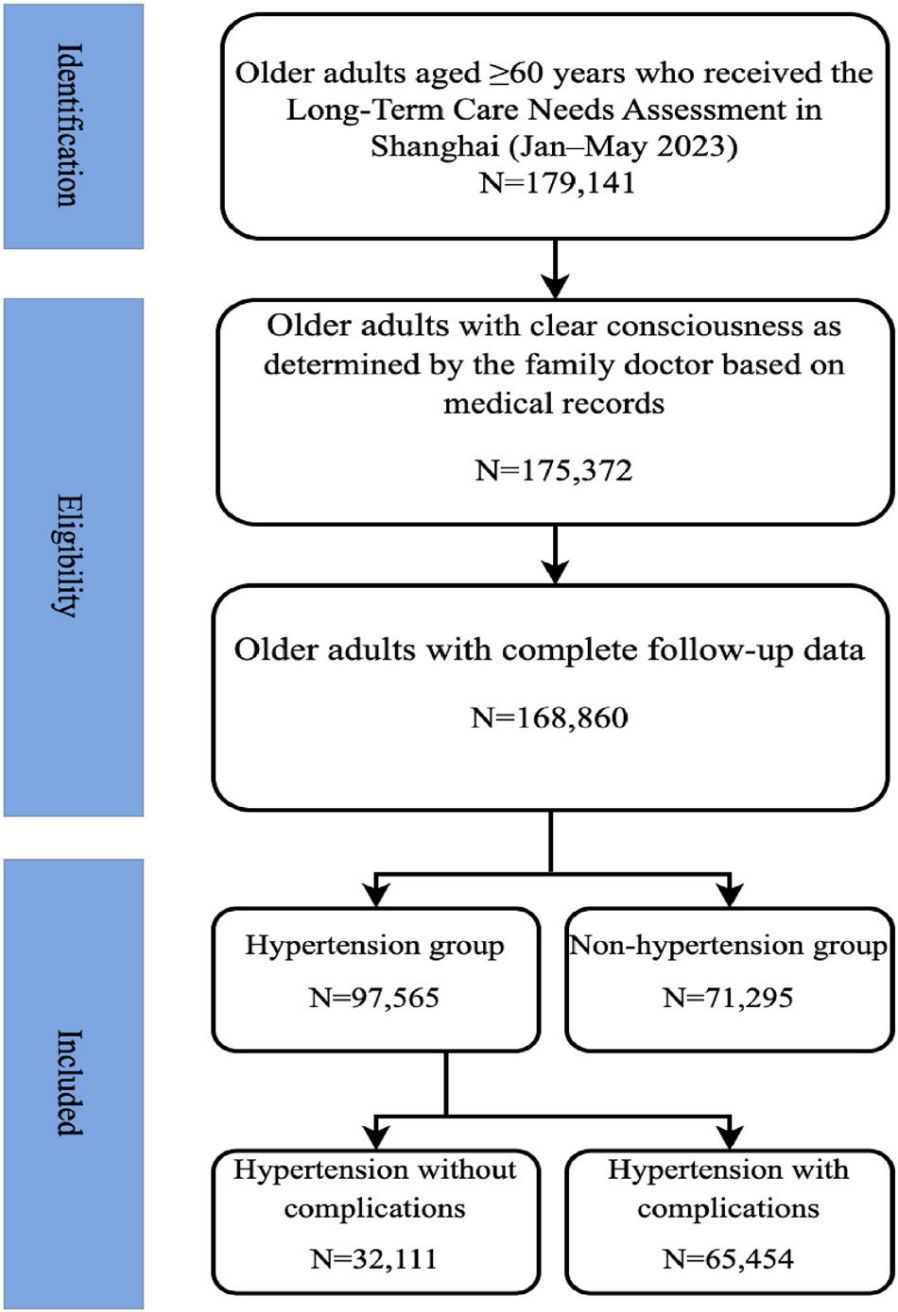
Flow chart of participants selection.

Initially, a total of 179,141 elderly adults were included. The average age was 80.8 ± 8.8 years, and 70,572 (39.4%) were male and 108,569 (60.6%) were female. A total of 3769 individuals with impaired consciousness and 6512 individuals with missing or abnormal data were excluded. Then, 3769 individuals with unclear consciousness were excluded. Finally, 168,860 individuals were included in the study. Hypertension was recorded by family doctors familiar with the individual's condition, using medical records from the past six months as supporting evidence. Hypertension complications in this study included cerebrovascular disease, hypertensive encephalopathy, heart failure, and renal insufficiency. Elderly individuals with at least one complication were classified into the hypertension with complications group. Based on the inclusion criteria, 97,565 individuals were assigned to the hypertension group and 71,295 to the non‐hypertension group. Among those with hypertension, 32,111 had no complications and 65,454 had at least one complication (Figure 1).

### 2.3 Data Collection

The data in this study were gathered from the Elderly Care Unified Needs Assessment for Long‐Term Care Insurance in Shanghai, China. Assessors were required to complete a standardized city‐wide training program before becoming qualified to conduct home visits. Each assessment team conducting home visits included at least two professionals: one with experience in elderly care, nursing, or social work, and the other a family doctor familiar with the elderly individual's health condition. During the assessment, the presence of a family member, caregiver, or guardian was required to help provide information and assist with the assessment. After the assessment, family doctors were responsible for quality control to ensure that the questionnaire data accurately reflected the elderly individuals' conditions. Dr. Ya Yang supervised the evaluation process by randomly cross‐checking family doctors' assessments with medical records.

### 2.4 Variable Selection

The present study included four categories of variables: demographic characteristics, lifestyle characteristics, emotional states, healthcare utilization and self‐management ability. Firstly, demographic data, including gender, age, education level, activities of daily living (ADL) were included to describe the characteristics of the research population. Secondly, lifestyle factors such as BMI, blood pressure, dietary habits (vegetarian, halal, low‐sugar, and low‐salt), daily activity indicators (frequency of going out, vision, joint mobility limitations, and cognition function) were included due to their known associations with hypertension (Zhao et al. 2024; di Raimondo et al. 2021). Thirdly, emotional state and sleep quality were assessed as key variables, with self‐rated health included as a subjective measure reflecting both emotional well‐being and physical health status of older adults (Cai et al. 2022; Qiu et al. 2024; Cohen et al. 2015; Han et al. 2020; Li and Shang 2021; Haghayegh et al. 2023; Wu et al. 2024). Finally, family‐related factors (residence, living status, family support) and healthcare‐related variables (use of oral medication, frequency of drug dispensing, frequency of outpatient visits, and need for assistance during medical appointments) were also important for patients with hypertension, according to previous studies (Nguyen et al. 2023; Abegaz et al. 2017).

### 2.5 Main Variables

The main variables consisted of some basic demographic variables and key target variables of the present study. Living condition‐related data were reported by elderly individuals themselves or their primary caregivers, while medical condition‐related data were assessed by family doctors familiar with the elderly person's circumstances, or derived from medical records. Living condition‐related data included gender, age, education level, and self‐rated health status. Self‐rated status was classified as “fairly well” and “poor”. Medical condition‐related data included blood pressure, body mass index (BMI), and activities of daily living (ADL). BMI was classified as underweight (< 18.5 kg/m^2^), normal weight (18.5–23.9 kg/m^2^), overweight (24.0–27.9 kg/m^2^) and obesity (≥ 28 kg/m^2^). ADL was assessed using a self‐design scale consisting of 20 items and 20 points in total. This scale was adapted from the activity of daily living scale designed by Lawton and Brody in 1969 (Lawton and Brody 1969). The cut‐off value for “Normal” group was 20.0 points, “Mild disability” group was 12.1‐19.9 points, “Moderate disability” group was 8.1–12.0 points, and “Severe disability” group was 0–8.0 points.

### 2.6 Other Variables

To understand the supplementary effects of other variables, some demographic characteristics were included: residence, living status, family support, cognitive function, vision, and joint activity limit. Residence was categorized as institutional care or home care, and living status was categorized as living alone or with others. Family support was based on the response to the question “What do you think of your family's support?,” and was classified as “good,” “moderate,” and “poor.” Cognition function, vision, and joint activity limit were assessed by family doctors familiar with the elderly person's circumstances or based on medical records. Joint mobility limitations was defined as a reduction in the range of motion by more than 50%. The affected joints included the cervical, shoulder, elbow, lumbar, hip, and knee joints.

Lifestyle characteristics included dietary habits and frequency of going out. Dietary habits included vegetarian (Yes/No), halal (Yes/No), low‐sugar (Yes/No), and low‐salt (Yes/No). Frequency of going out was classified as “rarely go out” (≤ 1 per month), “occasionally go out” (about once per week), and “frequently go out” (≥ 1 per day). Healthcare utilization and self‐management ability included oral medication, frequency of outpatient visits, frequency of drug dispensing, and accompanying medical treatment. Oral medication was classified as “completely self‐care” and “partial self‐care.” Frequency of outpatient visits and drug dispensing was based on medical records from the past 12 months, and accompanying medical treatment was defined as having at least one person accompany the elderly individual when attending medical appointments.

### 2.7 Assessment of Emotional State and Sleep Quality

Emotional state was self‐reported by older adults and recorded by the assessor, based on responses to the questions: “Have you felt more easily angered or agitated than usual in the past week?” and “Do you often feel fatigued for no apparent reason?” Responses were categorized as “never or seldom,” “sometimes,” and “often.” Sleep quality was assessed based on the response to the question: “How is your sleep quality at night?” and was classified as “good,” “moderate,” and “poor.”

### 2.8 Statistical Analysis

Data analysis was conducted using SPSS 23.0 software. First, a Chi‐square test (*χ^2^*) was used to compare demographic characteristics between the hypertension and non‐hypertension groups, as well as between those with and without complications among the hypertensive group. Next, a multivariate logistic regression model was conducted to analyze the impact of emotional state and sleep quality among all patients with hypertension and hypertension patients with complications. Finally, binary logistic regression was conducted to explore the possible interactions between emotional state and sleep quality. Based on the groups of emotional state and sleep quality, four models were included in this analysis: model 1 refers to “often feeling anger/agitation and with poor sleep quality,” model 2 refers to “often feeling anger/agitation and with moderate sleep quality,” model 3 refers to “sometimes feeling anger/agitation and with poor sleep quality,” and model 4 refers to “sometimes feeling anger/agitation and with moderate sleep quality.” Statistical significance was set at a *p*‐value of less than 0.05.

## 3 Results

### 3.1 General Information

Overall, significant differences were observed across variables including demographic characteristics, lifestyle, emotional state, healthcare utilization, and self‐management ability (Table 1, Table S1). The proportion of individuals with poorer emotional states (e.g., frequently feeling angered or fatigued) was significantly higher in the hypertension group than in the non‐hypertensive group (7.7% vs. 6.6%). Among elderly individuals with hypertension and complications, those with complications had a significantly higher proportion of poor emotional states compared to those without complications (8.8% vs. 5.3%). Additionally, the proportion of individuals with better sleep quality was significantly higher in the hypertension without complications group compared to the group with complications (44.7% vs. 29.1%). In the non‐hypertensive group, the gender ratio was relatively balanced between males and females, whereas in both the hypertension without complications and hypertension with complications groups, the proportion of males was significantly higher at 64.0% and 60.6%, respectively. Elderly individuals aged 80 and above constituted the highest proportion in the hypertension with complications group (60.0%), and also made up a substantial share of the hypertension without complications (58.8%) and non‐hypertensive groups (54.3%).

**TABLE 1:** Demographic characteristics of participants.

Characteristics	Hypertension group (*N* = 97,565)		Non‐hypertensio*n* group (*N* = 71,295)	χ^2*^	*P* ^*^	χ^2**^	*P* ^**^
	Without complications (*N* = 32,111)	With complications (*N* = 65,454)					
	*N* (%)	*N* (%)	*N* (%)				
Demographics							
Gender (Male)	20,556 (64.0%)	39,683 (60.6%)	42,010 (58.9%)	104.69	< 0.001	136.97	< 0.001
Age				48.98	< 0.001	861.81	< 0.001
61–69	3900 (12.1%)	6974 (10.7%)	11,175 (15.7%)				
70–79	9332 (29.1%)	19,184 (29.3%)	21,422 (30.0%)				
≥ 80	18,879 (58.8%)	39,296 (60.0%)	38,698 (54.3%)				
Education level				1576.18	< 0.001	198.39	< 0.001
Illiteracy	13,253 (41.3%)	19,324 (29.5%)	21,827 (30.6%)				
≤ 6 years	6905 (21.5%)	13,913 (21.3%)	15,077 (21.2%)				
7–12 years	9836 (30.6%)	26,036 (39.8%)	28,372 (39.8%)				
> 12 years	2096 (6.5%)	6076 (9.3%)	5979 (8.4%)				
BMI				24.73	< 0.001	1802.05	< 0.001
Normal weight	17,986 (56.0%)	35,631 (54.4%)	41,132 (58.0%)				
Underweight	3169 (9.9%)	6733 (10.3%)	10,860 (15.3%)				
Overweight	8128 (25.3%)	17,310 (26.4%)	14,801 (20.9%)				
Obesity	2651 (8.3%)	5512 (8.4%)	4089 (5.8%)				
Blood pressure				762.84	< 0.001	1688.60	< 0.001
Normal	17,013 (53.0%)	31,796 (48.6%)	71,295 (100.0%)				
141–159 mmHg/91‐99mmHg	11,190 (34.8%)	28,171 (43.0%)	—				
160–180 mmHg/100–110mmHg	3052 (9.5%)	4301 (6.6%)	—				
> 180 mmHg/110mmHg	856 (2.7%)	1186 (1.8%)	—				
ADL				4668.47	< 0.001	222.40	< 0.001
Normal	378 (1.2%)	364 (0.6%)	1038 (1.5%)				
Mild disability	13,545 (42.2%)	16,807 (25.7%)	21,441 (30.1%)				
Moderate disability	12,155 (37.9%)	23,034 (35.2%)	25,221 (35.4%)				
Severe disability	6033 (18.8%)	25,249 (38.6%)	23,595 (33.1%)				
Self‐rated health				4056.19	< 0.001	43.79	< 0.001
Fairly well	14,359 (44.7%)	16,107 (24.6%)	23,346 (32.7%)				
Poor	17,752 (55.3%)	49,347 (75.4%)	47,949 (67.3%)				
Lifestyle							
Sleep				2385.44	< 0.001	1674.10	< 0.001
Good	14,364 (44.7%)	19,056 (29.1%)	31,396 (44.0%)				
Moderate	17,342 (54.0%)	44,800 (68.4%)	38,772 (54.4%)				
Poor	405 (1.3%)	1598 (2.4%)	1127 (1.6%)				
Emotional state							
Anger/agitation				1829.28	< 0.001	247.01	< 0.001
Never or seldom	27,685(86.2%)	52,247(79.8%)	60,466 (84.8%)				
Sometimes	2719(8.5%)	7441(11.4%)	6134 (8.6%)				
Often	1707(5.3%)	5766(8.8%)	4695 (6.6%)				
Feeling fatigued					< 0.001	393.05	< 0.001
Never or seldom	12,529(39%)	18,604(28.4%)	25,550 (35.8%)				
Sometimes	4567(14.2%)	7647(11.7)	7309 (10.3%)				
Often	15,015(46.8%)	39,203(59.9)	38,436 (53.9%)				

*Note*: χ^2*^, *P* ^*^ represents the intergroup differences between elderly individuals with hypertension complications and those without complications.

χ^2**^, *P* ^*^ ^*^ represents the intergroup differences between elderly individuals with hypertension and those without hypertension.

BMI = Body Mass Index; ADL = Activities of Daily Living.

### 3.2 Factors Associated With Elderly Individuals With Hypertension and Complications

A multivariate logistic regression model was used to analyze the factors associated with elderly individuals with hypertension and complications (Table 2, Table S2, Table 3, Table S3). Males had a lower risk of hypertension (OR = 0.96), but a relatively higher risk of complications (OR = 1.11). The incidence of hypertension and complications increased significantly with age. Notably, individuals aged ≥ 80 had a higher risk of hypertension (OR = 1.51) and complications (OR = 1.20). Overall, hypertension patients with higher education levels (> 12 years) had a lower risk of hypertension (OR = 0.82; Table 2). However, among hypertensive individuals with complications, those with higher education levels were more likely to exhibit poorer health management outcomes (OR = 1.56; Table 3). This may suggest that individuals with higher educational backgrounds have earlier access to medical services, resulting in earlier detection of complications. Moreover, individuals being overweight or obese significantly increased the risk of hypertension, with obesity posing a greater risk (OR = 1.59). However, this relationship was not significant in the complications group.

**TABLE 2:** Multivariable logistic regression analysis on correlative factors of health management among hypertension patients.

Characteristics	OR	95%CI	*P*
Demographics			
Gender			
Female	1.00	—	—
Male	0.96	0.94–0.99	0.001
Age			
61–69	1.00	—	—
70–79	1.31	1.27–1.36	< 0.001
≥ 80	1.51	1.46–1.56	< 0.001
Education level			
Illiteracy	1.00	—	—
≤ 6 years	0.87	0.84–0.89	< 0.001
7–12 years	0.82	0.80–0.84	< 0.001
> 12 years	0.82	0.79–0.85	< 0.001
BMI			
Normal weight	1.00	—	—
Underweight	0.68	0.66–0.70	< 0.001
Overweight	1.36	1.32–1.39	< 0.001
Obesity	1.59	1.53–1.66	< 0.001
ADL			
Normal	1.00	—	—
Mild disability	1.26	1.13–1.40	< 0.001
Moderate disability	1.14	1.02–1.27	0.020
Severe disability	1.08	0.96–1.20	0.199
Self‐rated health			
Fairly well	1.00	—	—
Poor	0.96	0.94–0.99	0.002
Lifestyle			
Sleep			
Good	1.00	—	—
Moderate	1.51	1.48–1.55	< 0.001
Poor	1.67	1.55–1.81	< 0.001
Emotional state			
Anger/agitation			
Never or seldom	1.00	—	—
Sometimes	1.15	1.11–1.20	< 0.001
Often	1.18	1.14–1.23	< 0.001
Feeling fatigued			
Never or seldom	1.00	—	—
Sometimes	1.29	1.25–1.34	< 0.001
Often	1.03	1.01–1.06	0.011

BMI = Body Mass Index; ADL = Activities of Daily Living; OR = Odds Ratio; CI = Confidence Interval.

**TABLE 3:** Multivariable logistic regression analysis on correlative factors of health management among hypertension patients with complications.

Characteristics	OR	95%CI	*P*
**Demographics**			
Gender			
Female	1.00	—	—
Male	1.11	1.08–1.15	< 0.001
Age			
61–69	1.00	—	—
70–79	1.23	1.17–1.30	< 0.001
≥ 80	1.20	1.14–1.26	< 0.001
Education level			
Illiteracy	1.00	—	
≤ 6 years	1.20	1.15–1.25	< 0.001
7–12 years	1.53	1.47–1.60	< 0.001
> 12 years	1.56	1.47–1.66	< 0.001
BMI			
Normal weight	1.00	—	—
Underweight	0.89	0.85–0.94	< 0.001
Overweight	1.08	1.04–1.12	< 0.001
Obesity	1.04	0.98–1.10	0.18
Blood pressure			
Normal	1.00	—	—
141‐159 mmHg/91–99mmHg	1.25	1.21–1.29	< 0.001
160‐180 mmHg/100–110mmHg	0.86	0.81–0.91	< 0.001
> 180 mmHg/110mmHg	0.88	0.80–0.97	0.013
ADL			
Normal	1.00	—	—
Mild disability	1.13	0.96–1.33	0.139
Moderate disability	1.29	1.10–1.52	0.002
Severe disability	1.93	1.64–2.27	< 0.001
Self‐rated health			
Fairly well	1.18	1.10–1.27	< 0.001
Poor	1.64	1.42–1.90	< 0.001
Lifestyle			
Sleep			
Good	1.00	—	—
Moderate	1.34	1.29–1.38	< 0.001
Poor	1.38	1.23–1.56	< 0.001
Emotional state			
Anger/agitation			
Never or seldom	1.00	—	—
Sometimes	1.13	1.07–1.18	< 0.001
Often	0.98	0.92–1.04	0.473
Feeling fatigued			
Never or seldom	1.00	—	—
Sometimes	1.20	1.14–1.26	< 0.001
Often	1.29	1.25–1.34	< 0.001

BMI = Body Mass Index; ADL = Activities of Daily Living; OR = Odds Ratio; CI = Confidence Interval.

Sleep quality was significantly associated with both the hypertension and the complication groups. Individuals with poor sleep quality had a higher risk of hypertension in both groups (hypertension group OR = 1.67; complications group OR = 1.38), indicating a strong association between sleep disturbances and blood pressure regulation. Emotional states were also significantly associated with both the hypertension and the complication groups. The frequency of anger/agitation was positively associated with hypertension (sometimes: OR = 1.15; often: OR = 1.18). In the complications group, individuals who sometimes experienced anger/agitation were also more likely to have complications (OR = 1.13). The frequency of feeling fatigue was positively associated with hypertension (sometimes: OR = 1.29; often: OR = 1.03), and significant correlations were also observed in the complications group (sometimes: OR = 1.20, often: OR = 1.29).

### 3.3 Interaction of Emotion and Sleep Conditions

To explore the interaction between emotion state and sleep quality, a binary logistic regression model was conducted (Table 4). The results indicated that individuals who sometimes experienced anger/agitation and had moderate sleep quality were significantly more likely to have hypertension (OR = 1.35). Moreover, compared to individuals who sometimes experienced anger/agitation and had poor sleep quality (OR = 1.27), those who often experienced anger/agitation and had moderate sleep quality exhibited the highest risk of hypertension (OR = 1.44). Notably, individuals who sometimes experienced anger/agitation and had moderate sleep quality were significantly associated with a higher risk of complications (OR = 1.15), as were those who often experienced anger or agitation with moderate sleep quality (OR = 1.14). However, among individuals who often experienced anger/agitation and had poor sleep quality, no significant association was observed in the hypertension or the complications groups (*P* > 0.05).

**TABLE 4:** Analysis on interaction of emotion and sleep conditions.

Interactive Model	Hypertension			Hypertension with complications		
	OR	95%CI	*P*	OR	95%CI	*P*
Reference model	1.00	—	—	1.00	—	—
Model 1	1.06	0.85–1.31	0.632	1.28	0.84–1.96	0.252
Model 2	1.44	1.37–1.51	< 0.001	1.14	1.06–1.23	< 0.001
Model 3	1.27	1.01–1.59	0.041	0.9	0.65–1.25	0.529
Model 4	1.35	1.30–1.41	< 0.001	1.15	1.09–1.22	< 0.001

*Note*: Reference model: Never or seldom feeling anger/agitation and with good sleep quality.

Model 1: Often feeling anger/agitation and with poor sleep quality.

Model 2: Often feeling anger/agitation and with moderate sleep quality.

Model 3: Sometimes feeling anger/agitation and with poor sleep quality.

Model 4: Sometimes feeling anger/agitation and with moderate sleep quality.

OR = Odds Ratio; CI = Confidence Interval.

## 4 Discussion

This study, based on a large‐scale cross‐sectional database of 168,860 older adults in Shanghai, explored the relationship between emotional state, sleep quality, and the risk of hypertension and its complications. Among the 97,565 individuals diagnosed with hypertension, 65,454 had at least one comorbidity. Results revealed that emotional instability (e.g., irritability and fatigue) and poor sleep quality were more serious among individuals with hypertension, particularly those with complications, compared to individuals without hypertension. This study found a notable interaction effect: individuals with moderate emotional disturbances and moderate sleep problems may have the highest odds ratio for developing complications. These findings suggest that emotional and sleep‐related factors may synergistically contribute to heightened cardiovascular risk in older populations.

This study presents several notable strengths. First, the large and representative sample significantly enhances statistical power and external validity. All participants were assessed through Shanghai's unified long‐term care insurance evaluation system, ensuring standardized data collection and reliability. Second, the present study focused on patients with hypertensive complications and analyzed this population as separate subgroups, which has not been fully explored in previous studies. Third, this study provides a novel perspective by examining the interaction between emotional status and sleep quality in the context of complex geriatric health conditions. Lastly, real‐world data enhances the practical relevance of the findings, providing valuable insights for policy‐making in elderly healthcare in China.

The link between emotional health and hypertension has been extensively documented. Negative emotional states such as anxiety, depression, and irritability, are significantly associated not only with the onset of hypertension but also with its control rates, risk of complications, and overall quality of life. These effects are thought to occur through multiple physiological pathways, such as sympathetic nervous system activation, adrenal medullary hormone release, endothelial dysfunction, and systemic inflammation (Fan and Zhang 2023; Gan et al. 2023; Chinese Hypertension League 2025; Su et al. 2024). Additionally, studies have shown that emotional distress can markedly reduce medication adherence, increase blood pressure variability, and elevate the risk of target organ damage (Shen and Chen 2021). Emotional disorders are also frequently comorbid with unhealthy lifestyle behaviors, such as physical inactivity and poor diet, creating a vicious cycle of mutual reinforcement.

Similarly, sleep disorders including insomnia, short sleep duration, and frequent nighttime awakenings are recognized as independent risk factors for hypertension. Proposed mechanisms include excessive sympathetic activity, non‐dipping nocturnal blood pressure, dysregulation of the hypothalamic pituitary adrenal axis, and abnormal melatonin secretion (Li et al. 2023; Ma et al. 2024; Nemcsik‐Bencze et al. 2022; Liu et al. 2017; Liu et al. 2020).

The 2024 Chinese guidelines for the prevention and treatment of hypertension recommend routine assessment of sleep quality as part of initial screening and follow‐up (Shen and Chen 2021). Regional studies have also demonstrated a strong positive association between sleep issues (e.g., snoring, awakenings, sleep deprivation) and hypertension prevalence in middle‐aged and older adults in Shanghai (Guo et al. 2013; Sofi et al. 2014). Moreover, some anti‐hypertensive drugs such as β‐blockers and thiazide diuretics may adversely affect sleep by inducing insomnia, altering sleep architecture, or increasing nocturnal urination, thereby exacerbating emotional instability and impairing daytime functioning (Walsh et al. 2020; Yazdanpanah et al. 2019). Emerging research underscores the importance of assessing the potential sleep‐related side effects of medications and adjusting treatment plans accordingly (Bosworth et al. 2008). This study further revealed that even subclinical levels of emotional disturbance and poor sleep quality can exert a synergistic impact when they co‐occur; emphasizing the cumulative burden of psychosocial stressors. This aligns with the growing adoption of multifactorial risk models in chronic disease management, advocating for the integration of psychological and sleep interventions in hypertension control, especially for older patients with complications (Chen et al. 2008; Desalegn et al. 2021).

These findings have significant implications for comprehensive prevention strategies. While the independent effects of emotional and sleep disturbances on hypertension are well established, their combined influence suggests that dual‐domain interventions may be more effective than single‐focus strategies. Examples include cognitive behavioral therapy (CBT) for insomnia, emotion regulation training, and integrated psycho‐sleep management models (Poulter et al. 2020; Irwin 2019). Embedding mental health services into primary care systems has shown promising results in chronic disease management (Burnier et al. 2020). Considering that many elderly patients may underreport emotional or sleep‐related symptoms due to communication difficulties or stigma, routine screening for these factors in primary care is highly recommended. Moreover, the heightened risk observed in individuals with comorbid complications calls for targeted, individualized intervention pathways for high‐risk subgroups. These insights resonate with global trends in chronic disease prevention and advocate for the inclusion of psychosocial variables in cardiovascular risk assessment models to promote multidisciplinary, personalized prevention frameworks (Kuo 2019; Zhang et al. 2022).

There were also several limitations of this study. First, this study used a cross‐sectional design, which may limit the strength of causal inferences. However, the large sample size enhances the reliability of the results and supports their generalizability to the target population. Second, this study used a self‐reported questionnaire, which may introduce reporting bias, particularly related to participants’ education levels. Another cross‐sectional study also revealed a connection between education level and the knowledge and attitudes related to self‐care in the management of chronic diseases (Borba et al. 2019). Therefore, measures were taken to reduce this bias in the present study, such as quality control performed by family doctors.

## 5 Conclusion

This cross‐sectional study included a large study population of 168,860 older adults. It explored the relationship between emotional state, sleep quality, and the risk of hypertension and its complications. Emotional instability and poor sleep quality were more pronounced among individuals with hypertension, especially those with complications. Moreover, an interaction effect indicated that individuals with moderate emotional disturbances and moderate sleep problems may have the highest odds ratio for developing complications. These findings contribute important epidemiological evidence from older adults in China and support the development of targeted prevention strategies for hypertension and its complications.

## Author Contributions

**Jin Wang**: conceptualization, methodology, data curation, project administration, writing ‐ review and editing, writing ‐ original draft, funding acquisition, validation, visualization, formal analysis. **Yunwei Zhang**: writing ‐ original draft, writing ‐ review and editing, conceptualization, data curation, software, investigation. **Yiman Feng**: writing ‐ original draft. **Ya Yang**: writing ‐ review and editing, resources, data curation. **Hansheng Ding**: writing ‐ review and editing, project administration, resources, data curation, software, supervision.

## Conflicts of Interest

The authors declare no conflicts of interest

## Peer Review

The peer review history for this article is available at https://publons.com/publon/10.1002/brb3.70676

## Supporting information




**Supporting Table S1**: Demographic characteristics of participants (other variables)
**Supporting Table S2**: Multivariable logistic regression analysis on correlative factors of health management among hypertension patients (other variables)
**Supporting Table S3**: Multivariable logistic regression analysis on correlative factors of health management among hypertension patients with complications (other variables)

## Data Availability

The data that support the findings of this study are available on request from the corresponding author. The data are not publicly available due to privacy or ethical restrictions.
