# Supplementary material for: Association of Emotion, Sleep Quality With Hypertension and Complications in the Elderly Population
Source: Brain Behav. 2025 Jul 7;15(7):e70676. doi: 10.1002/brb3.70676 (PMC12230633; doi:10.1002/brb3.70676)
Supplement: Supplementary file 1 — Supporting Table S1: Demographic characteristics of participants (other variables) Supporting Table S2: Multivariable logistic regression analysis on correlative factors of health management among hypertension patients (other variables) Supporting Table S3: Multivariable logistic regression analysis on correlative factors of health management among hypertension patients with complications (other variables) [file BRB3-15-e70676-s001.docx]

Supplementary Table S1–S3 provided extended descriptive and regression results referred to in the main text (see Methods)

**Supplementary material**

**Table S1. Demographic characteristics of participants (other variables)**

| Characteristics | Hypertension group  (N=97,565） | | Non-hypertension group  (N=71,295) | *χ^2*^* | *P*^*^ | *χ^2**^* | *P*^**^ |
| --- | --- | --- | --- | --- | --- | --- | --- |
|  | Without complications  (N=32,111) | With complications  (N=65,454) |  |  |  |  |  |
|  | N(%) | N(%) | N(%) |  |  |  |  |
| **Demographics** | | | | | | | |
| Residence | | |  | 90.85 | ＜0.001 | 221.10 | ＜0.001 |
| Institutional care | 2582 (8.0%) | 6498 (9.9%) | 8219 (11.5%) |  |  |  |  |
| Home care | 29529 (92.0%) | 58956 (90.1%) | 63076 (88.5%) |  |  |  |  |
| Living status (alone) | 3876 (12.1%) | 6819 (10.4%) | 7660 (10.7%) | 60.28 | ＜0.001 | 2.02 | 0.016 |
| Family support | | |  | 414.29 | ＜0.001 | 102.72 | ＜0.001 |
| Good | 11214 (34.9%) | 20205 (30.9%) | 24146 (33.9%) |  |  |  |  |
| Moderate | 15819 (49.3%) | 31513 (48.1%) | 32813 (46.0%) |  |  |  |  |
| Poor | 5078 (15.8%) | 13736 (21.0%) | 14336 (20.1%) |  |  |  |  |
| Cognition function | | | | 4227.72 | ＜0.001 | 628.97 | ＜0.001 |
| Normal | 26433 (82.3%) | 40640 (62.1%) |  |  |  |  |  |
| MCI | 5449 (17.0%) | 22435 (34.3%) |  |  |  |  |  |
| Dementia | 229 (0.7%) | 2379 (3.6%) |  |  |  |  |  |
| Vision (Normal) | 11810 (36.8%) | 12547 (19.2%) | 24534 (34.4%) | 3566.11 | ＜0.001 | 1787.21 | ＜0.001 |
| Joint activity limit (Yes) | 8572 (26.7%) | 23207 (35.5%) | 21975 (30.8%) | 752.36 | ＜0.001 | 58.19 | ＜0.001 |
| **Lifestyle** | | | | | | | |
| Vegetarian (Yes) | 1770 (5.5%) | 4614 (7.0%) | 4826 (6.8%) | 83.23 | ＜0.001 | 3.39 | 0.070 |
| Halal (Yes) | 269 (0.8%) | 1370 (2.1%) | 932 (1.3%) | 205.54 | ＜0.001 | 38.15 | ＜0.001 |
| Low-sugar (Yes) | 7503 (23.4%) | 20588 (31.5%) | 16981 (23.8%) | 687.4 | ＜0.001 | 520.87 | ＜0.001 |
| Low-salt (Yes) | 25194 (78.5%) | 47135 (72.0%) | 52447 (73.6%) | 466.90 | ＜0.001 | 6.96 | 0.010 |
| Frequency of going out | | | | 67.76 | ＜0.001 | 175.4 | ＜0.001 |
| Rarely go out | 28526 (88.8%) | 58889 (90.0%) | 64396 (90.3%) |  |  |  |  |
| Occasionally go out | 2749 (8.6%) | 5365 (8.2%) | 4951 (6.9%) |  |  |  |  |
| Frequently go out | 836 (2.6%) | 1200 (1.8%) | 1948 (2.7%) |  |  |  |  |
| **Healthcare utilization and self-management ability** | | | | | | | |
| Oral medication | | | | 1110.46 | ＜0.001 | 186.29 | ＜0.001 |
| Completely self-care | 8348 (26.0%) | 24013 (36.7%) | 25927 (36.4%) |  |  |  |  |
| Partial self-care | 23763 (74.0%) | 41441 (63.3%) | 45368 (63.6%) |  |  |  |  |
| Frequency of drug dispensing | | | | 378.03 | ＜0.001 | 151.95 | ＜0.001 |
| Less than once per month | 10435 (32.5%) | 18635 (28.5%) | 22931 (32.2%) |  |  |  |  |
| 1-2 times a month | 18819 (58.6%) | 38920 (59.5%) | 41441 (58.1%) |  |  |  |  |
| 2-3 times a month | 2069 (6.4%) | 5038 (7.7%) | 4518 (6.3%) |  |  |  |  |
| More than 3 times per month | 788 (2.5%) | 2861 (4.4%) | 2405 (3.4%) |  |  |  |  |
| Frequency of outpatient visits | | | | 341.42 | ＜0.001 | 70.04 | ＜0.001 |
| Less than once per month | 26377 (82.1%) | 50910 (77.8%) | 56398 (79.1%) |  |  |  |  |
| 1-2 times a month | 4848 (15.1%) | 11397 (17.4%) | 12390 (17.4%) |  |  |  |  |
| 2-3 times a month | 464 (1.4%) | 1733 (2.6%) | 1234 (1.7%) |  |  |  |  |
| More than 3 times per month | 422 (1.3%) | 1414 (2.2%) | 1273 (1.8%) |  |  |  |  |
| Accompanying medical treatment (Yes) | 26226 (81.7%) | 50731 (77.5%) | 55289 (77.5%) | 224.47 | ＜0.001 | 42.78 | ＜0.001 |

Note: *χ^2*^*, *P*^*^ represents the intergroup differences between elderly individuals with hypertension complications and those without complications.

*χ^2**^*, *P*^*^*^*^* represents the intergroup differences between elderly individuals with hypertension and those without hypertension.

**Table S2. Multivariable logistic regression analysis on correlative factors of health management among hypertension patients (other variables)**

| Characteristics | *OR* | *95%CI* | *P* |
| --- | --- | --- | --- |
| **Demographics** | | | |
| Residence | | |  |
| Institutional care | 1.00 | - | - |
| Home care | 1.33 | 1.28-1.37 | ＜0.001 |
| Living status | | | |
| Alone | 1.00 | - | - |
| With others | 0.99 | 0.96-1.03 | 0.757 |
| Family support | | |  |
| Good | 1.00 | - | - |
| Moderate | 1.04 | 1.01-1.07 | 0.001 |
| Poor | 1.00 | 0.96-1.03 | 0.087 |
| Cognition function | | | |
| Normal | 1.00 | - | - |
| MCI | 0.93 | 0.88-0.99 | 0.026 |
| Dementia | 1.24 | 1.21-1.27 | ＜0.001 |
| Vision | | | |
| Normal | 1.00 | - | - |
| Abnormal | 1.53 | 1.50-1.59 | ＜0.001 |
| Joint activity limit | | | |
| No | 1.00 | - | - |
| Yes | 0.99 | 0.97-1.02 | 0.493 |
| **Lifestyle** | | | |
| Vegetarian | | | |
| No | 1.00 | - | - |
| Yes | 1.03 | 0.99-1.08 | 0.157 |
| Halal | | | |
| No | 1.00 | - | - |
| Yes | 1.24 | 1.13-1.36 | ＜0.001 |
| Low-sugar | | | |
| No | 1.00 | - | - |
| Yes | 1.28 | 1.25-1.31 | ＜0.001 |
| Low-salt | | | |
| No | 1.00 | - | - |
| Yes | 1.08 | 1.05-1.11 | ＜0.001 |
| Frequency of going out | | | |
| Rarely go out | 1.00 | - | - |
| Occasionally go out | 1.15 | 1.11-1.20 | ＜0.001 |
| Frequently go out | 1.04 | 0.96-1.11 | 0.352 |
| **Healthcare utilization and self-management ability** | | | |
| Oral medication | | | |
| Completely self-care | 1.00 | - | - |
| Partial self-care | 1.08 | 1.06-1.11 | ＜0.001 |
| Frequency of drug dispensing | | | |
| Less than once per month | 1.00 | - | - |
| 1-2 times a month | 1.07 | 1.02-1.12 | 0.005 |
| 2-3 times a month | 1.07 | 1.02-1.12 | 0.005 |
| More than 3 times per month | 1.08 | 1.02-1.15 | 0.010 |
| Frequency of outpatient visits | | | |
| Less than once per month | 1.00 | - | - |
| 1-2 times a month | 0.89 | 0.87-0.92 | ＜0.001 |
| 2-3 times a month | 1.23 | 1.14-1.33 | ＜0.001 |
| More than 3 times per month | 1.01 | 0.93-1.09 | 0.860 |
| Accompanying medical treatment | | | |
| No | 1.00 | - | - |
| Yes | 1.02 | 1.00-1.05 | 0.087 |

**Table S3. Multivariable logistic regression analysis on correlative factors of health management among hypertension patients with complications (other variables)**

| Characteristics | *OR* | *95%CI* | *P* |
| --- | --- | --- | --- |
| **Demographics** | | | |
| Residence | | |  |
| Institutional care | 1.00 | - | - |
| Home care | 1.29 | 1.22-1.36 | ＜0.001 |
| Living status | | | |
| Alone | 1.00 | - | - |
| With others | 0.89 | 0.85-0.94 | ＜0.001 |
| Family support | | |  |
| Good | 1.00 | - | - |
| Moderate | 1.10 | 1.06-1.14 | ＜0.001 |
| Poor | 1.18 | 1.13-1.24 | ＜0.001 |
| Cognition function | | | |
| Normal | 1.00 | - | - |
| MCI | 2.14 | 2.06-2.22 | ＜0.001 |
| Dementia | 4.13 | 3.58-4.76 | ＜0.001 |
| Vision | | | |
| Normal | 1.00 | - | - |
| Abnormal | 1.86 | 1.80-1.92 | ＜0.001 |
| Joint activity limit | | | |
| No | 1.00 | - | - |
| Yes | 1.03 | 1.00-1.07 | 0.062 |
| **Lifestyle** | | | |
| Vegetarian | | | |
| No | 1.00 | - | - |
| Yes | 1.15 | 1.11-1.19 | ＜0.001 |
| Halal | | | |
| No | 1.00 | - | - |
| Yes | 0.85 | 0.82-0.89 | ＜0.001 |
| Low-sugar | | | |
| No | 1.00 | - | - |
| Yes | 1.00 | 0.91-1.11 | 0.964 |
| Low-salt | | | |
| No | 1.00 | - | - |
| Yes | 1.07 | 1.03-1.12 | 0.001 |
| Frequency of going out | | | |
| Rarely go out | 1.00 | - | - |
| Occasionally go out | 1.05 | 0.99-1.10 | 0.104 |
| Frequently go out | 1.18 | 1.04-1.33 | 0.008 |
| **Healthcare utilization and self-management ability** | | | |
| Oral medication | | | |
| Completely self-care | 1.00 | - | - |
| Partial self-care | 0.89 | 0.85-0.92 | ＜0.001 |
| Frequency of drug dispensing | | | |
| Less than once per month | 1.00 | - | - |
| 1-2 times a month | 0.84 | 0.81-0.87 | ＜0.001 |
| 2-3 times a month | 0.79 | 0.74-0.85 | ＜0.001 |
| More than 3 times per month | 0.96 | 0.88-1.06 | 0.413 |
| Frequency of outpatient visits | | | |
| Less than once per month | 1.00 | - | - |
| 1-2 times a month | 1.07 | 1.03-1.12 | 0.001 |
| 2-3 times a month | 1.27 | 1.14-1.43 | ＜0.001 |
| More than 3 times per month | 1.18 | 1.04-1.33 | 0.008 |
| Accompanying medical treatment | | | |
| No | 1.00 | - | - |
| Yes | 0.78 | 0.75-0.81 | ＜0.001 |
